# The use of gamification in the teaching of disease epidemics and pandemics

**DOI:** 10.1093/femsle/fny111

**Published:** 2018-04-26

**Authors:** L A Robinson, I J Turner, M J Sweet

**Affiliations:** 1 Environmental Sustainability Research Centre, University of Derby, Derby, DE22 1GB, UK; 2 Centre for Excellence in Learning and Teaching, University of Derby, Derby, DE22 1GB, UK

**Keywords:** gamification, disease, epidemic, modelling, plague

## Abstract

With the launch of the teaching excellence framework, teaching in higher education (HE) is under greater scrutiny than ever before. Didactic lecture delivery is still a core element of many HE programmes but there is now a greater expectation for academics to incorporate alternative approaches into their practice to increase student engagement. These approaches may include a large array of techniques from group activities, problem-based learning, practical experience and mock scenarios to newly emerging approaches such as flipped learning practices and the use of gamification. These participatory forms of learning encourage students to become more absorbed within a topic that may otherwise be seen as rather ‘dry’ and reduce students engagement with, and therefore retention of, material. Here we use participatory-based teaching approaches in microbiology as an example to illustrate to University undergraduate students the potentially devastating effects that a disease can have on a population. The ‘threat’ that diseases may pose and the manner in which they may spread and/or evolve can be challenging to communicate, especially in relation to the timescales associated with these factors in the case of an epidemic or pandemic.

## Teaser

Highlighting how games can be used in higher education teaching to illustrate more complex fields of study.

**Figure 1.**
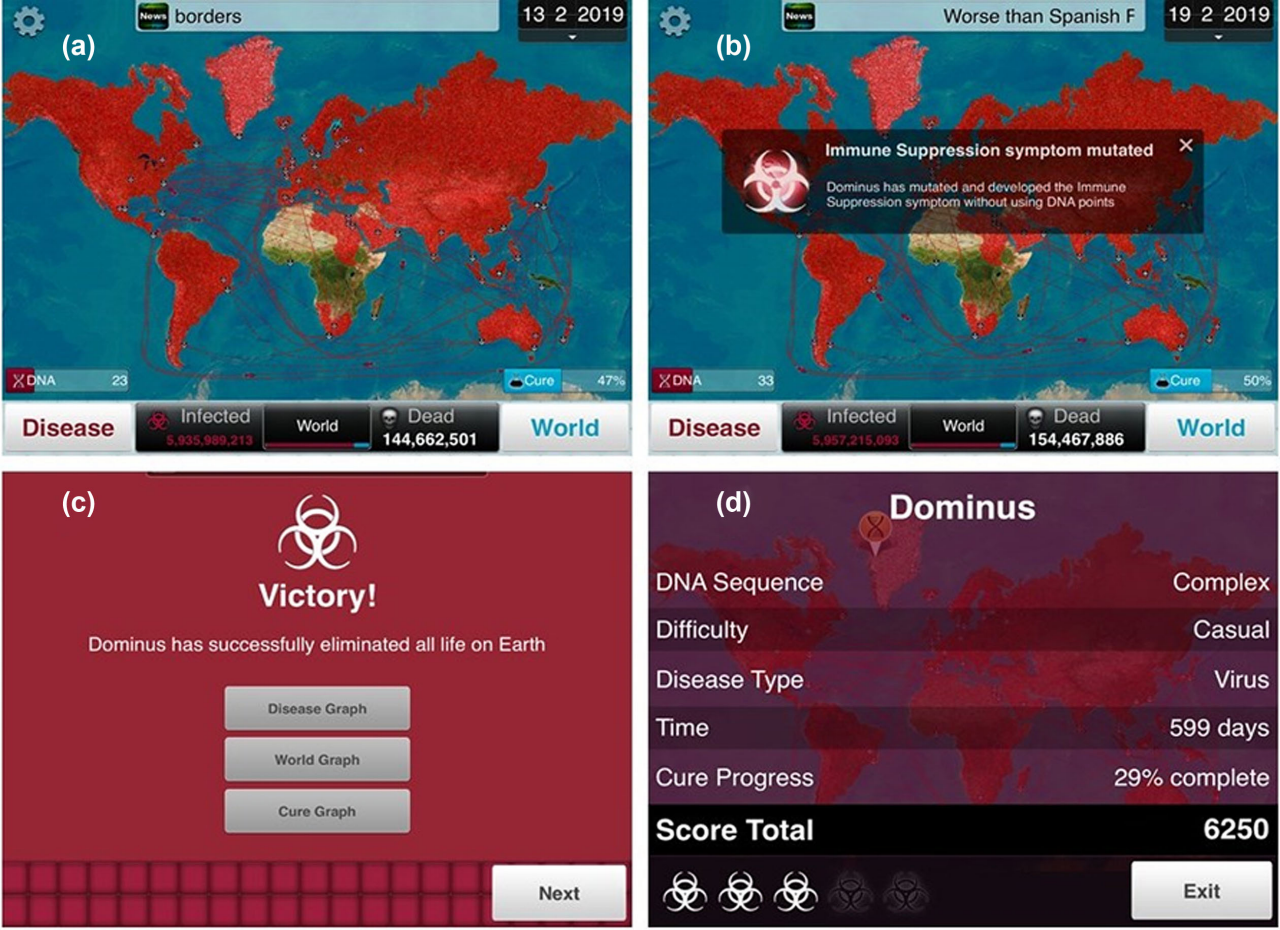
(**a**) Plague Inc. in progress showing the spread of disease, number of dead and infected, routes of transmission and cure progression. (**b**) Example of automatic evolution of the pathogen ‘immune suppression’. (**c**) Victory message if the player is successful in destroying all life on earth with options to view graphical displays of the progress of the pathogen. (**d**) Results after completion of the game.

The term ‘gamification’ is typically used to describe the application of game mechanics or motivational techniques in a non-game environment (Lee and Hammer 2011). Gamification has gained popularity in recent years in the education sector, having stemmed from approaches within business and marketing to improve customer experience and loyalty. It has been used successfully in further education environments, but more recently such approaches have been evidenced in HE (Kasurinen and Knutas 2018). The application of gamification in education is diverse, ranging from the use of simple game mechanics within a teaching session (i.e. how games work), up to and including the design and use of novel board or computer games. One intermediary way of introducing games into teaching is the utilisation of publically accessible educational games for the purpose of reinforcing a particular topic or learning outcome. However, when using physical board games, there is sometimes a challenge in accessing sufficient copies to support a larger class. An alternative approach would therefore be the utilisation of application-based games as they are less restrictive in their purchase price. One example that can be used in an HE setting and is available via multiple platforms is Plague Inc. (Ndemic Creations, UK). The game can be played on mobile devices when downloaded via the app store for a nominal cost (79p as of February 2018) or for free if utilising the Android version. Alternately, the game can also be purchased via the digital distribution platform ‘Steam’ for use on a PC—if mobile devices are not readily available. Plague Inc. (also available as a board game) is a strategic simulation-based game in which you as the player seek to decimate the global population by controlling a pathogen and selecting its specific traits such as transmission and symptoms. Humanity fights against the pathogen through the development of a cure that you monitor as a progressing percentage value (see Fig. 1a–d). In order to stay ahead, you find ways to strengthen your pathogen and increase its infection rate with the goal of wiping out the population before the cure can be developed.

Plague Inc. has been used within a level five (stage two) undergraduate Microbiology module at the University of Derby, UK, in a session that has learning outcomes related to the understanding of epidemics, pandemics and disease transmission. In the session, the students are given a broad introduction to the topic before playing the game twice. The launch of the first attempt at the game is typically followed by excitement and student eagerness to successfully ‘kill’ the entire population of the world. In the first attempt, students are unrestricted in the way they play and therefore see varying levels of success that can be discussed with the instructor as to reasons why. After this first round, students are asked a series of reflective questions for example: What have they just done? How did they infect the populace? Who was more effective at creating a ‘perfect’ pathogen and why? How did they evolve their pathogen? Where did they start their outbreak and why? In the second attempt, the students take a structured approach whereby the instructor guides groups of students into asking specific questions by utilising the in-game variables such as the most effective disease vector. At the end of the game, students report their results, in particular the number of ‘infected’ and those that are ‘dead’. The instructor then uses these figures to facilitate a discussion about the specific variables, i.e. vectors in this example. The students can then explore how other variables such as starting country of the disease outbreak (linked to population and transport routes) and/or modes of transmission can also be explored. If time permits, these can be tested through further rounds of the game or expanded into a directed study activity to be continued out of formal teaching hours.

Johnson (2006) stated that all games, not just those used for education, should encourage a scientific method of probing the environment, forming a hypothesis, re-probing to check the effect and re-thinking based on feedback. Plague Inc. is a perfect example of how this process can be utilised within a classroom setting in which students can develop a scientific approach and test it out in real time for further discussion. However, Plague Inc. does have a limitation in that it is programmed to automatically evolve as a ‘reward’ for creating a successful pathogen. To compensate this, these automatic ‘upgrades’ (for example antibiotic resistance) must be ‘de-evolved’ by the player to ensure a fair ‘experiment’. Any doubts about the validity of the results can be addressed by the instructor highlighting the importance of replication. Students further gain an understanding of stocasticity i.e. that random probability distributions or patterns can be analysed statically but are not always useful for precise predictions. With regard to disease epidemics—it is particularly important that students understand that in the Plague Inc. scenarios, many variables can be controlled that is unlike a real-world scenario. We can end these sessions with the instructor illustrating how programme coding can be used in disease modelling. For example, the Ebola outbreak of 2014–16 (Legrand *et al.*^2007^; Gomes *et al.*^2014^) and/or the spread of the Zika virus (Messina *et al.*^2016^) have been used in previously run sessions. The link between disease modelling is then drawn to how government bodies such as the World Health Organisation (WHO) or the Centre for Disease Control (CDC) can use such information in their management and mitigation strategies.

The advantages of mobile applications such as Plague Inc. are that they are free or cheap to utilise and provide a different form of media from which the student can learn. The setup of tablet devices (if available for student use) is minimal, and the cost of purchasing games is low. An alternative approach is to ‘bring your own device’ and ask students to install the application themselves (especially if they are to work in pairs or groups). This approach does have the risk of excluding students without the required technology or who are unwilling to purchase the application on their personal device, but this issue can be somewhat negated with the use of group work.

**Figure 2.**
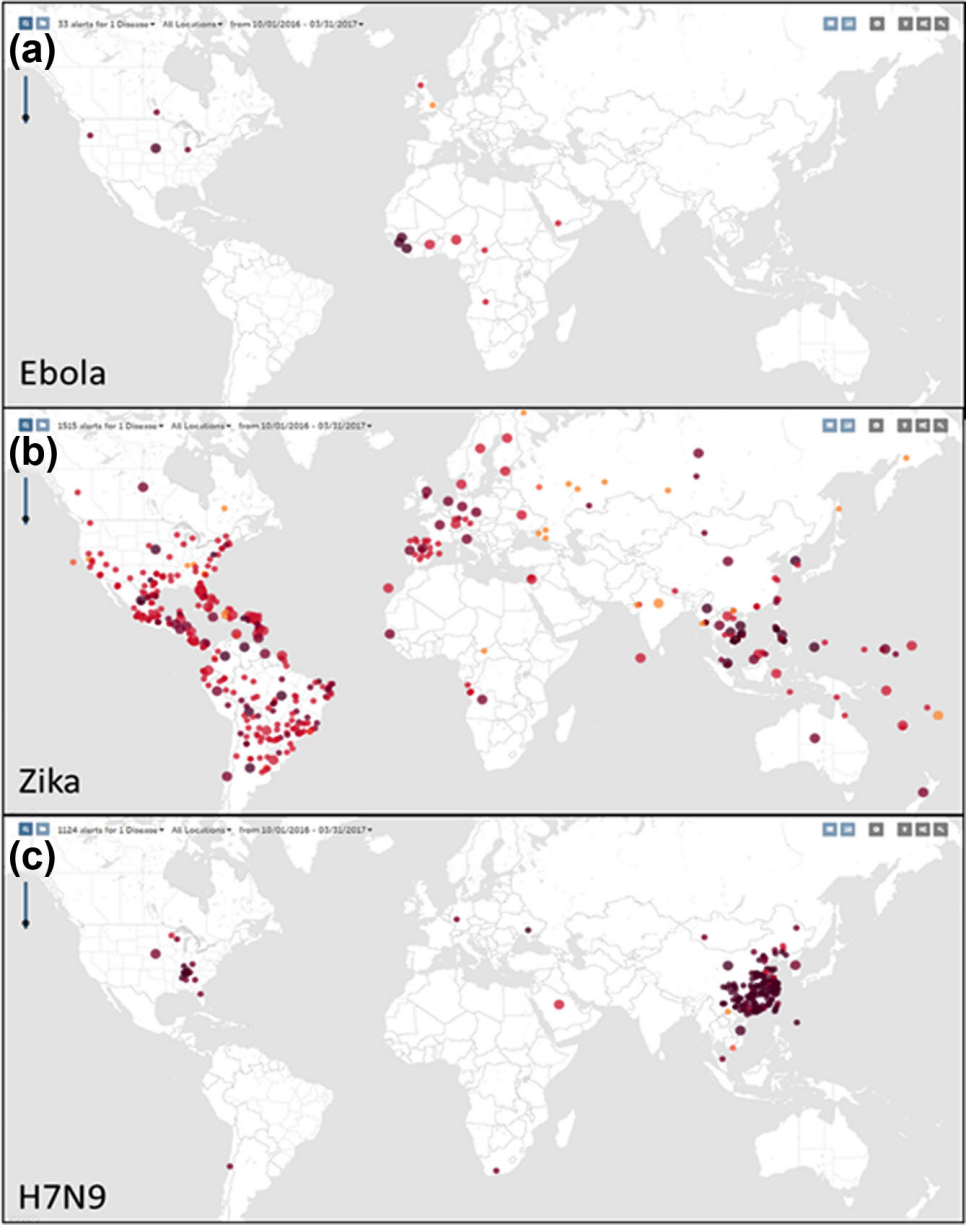
Representative images from healthmap.org/en/showing the reported case of (**a**) Ebola, (**b**) Zika and (**c**) H7N9.

Two tools that can also be used to enhance teaching in these classes are the Humanitarian Open StreetMap Tasking Manager and HeatMap.org. During the outbreak of Ebola in 2014–2016, the WHO declared a public emergency and other agencies such as the American Red Cross encouraged volunteers to map out particular areas that were data deficient (using the Humanitarian Open StreetMap Tasking Manager). The aim was to aid relief workers on the ground to reach out to isolated areas. This approach was combined with the use of HeatMap.org that allows viewers to track infectious diseases on a global scale. This interactive web interface allows anyone to explore which diseases are impacting or have impacted their area (Fig. 2). Utilising these sites, it was possible to track the outbreak of Ebola. Researchers and medical professionals were therefore able to confirm where the first signs and initial outbreak occurred. The combination of utilising such data, with simulated modelling of disease spread (i.e. from Plague Inc.) students can gain a greater understanding of the nature of disease epidemics and pandemics and the importance of accurately modelling such outbreaks with regard to management and mitigation. Learning outcomes from sessions such as these, mean students leave with (a) a clear understanding of factors governing disease outbreaks, (b) basic principles of disease modelling and (c) how such models can be utilised by management agencies in order to prevent or control such epidemics from occurring or remain restricted in their impact.

***Conflict of interest.*** None declared.
